# AquAdvantage Salmon - a pioneering application of biotechnology in aquaculture

**DOI:** 10.1186/1753-6561-8-S4-O31

**Published:** 2014-10-01

**Authors:** Henry Clifford

**Affiliations:** 1 AquaBounty Technologies, San Diego, United States

## 

The U.S. FDA is currently reviewing AquaBounty Technologies' New Animal Drug Application for a fast growing transgenic Atlantic salmon, known as AquAdvantage^® ^Salmon, which would be the first genetically modified food animal approved for human consumption. AquAdvantage^® ^Salmon was developed by inserting into a fertilized Atlantic salmon egg, a transgene constructed from the growth hormone gene of a related species (Chinook salmon). The AquAdvantage^® ^genetic construct confers upon the Atlantic salmon a rapid growth phenotype and improved metabolic efficiencies associated with low but sustained levels of growth hormone. This allows the fast growing AquAdvantage^® ^Salmon to reach market size in half the time of a conventional Atlantic salmon. AquAdvantage^® ^Salmon will be available only as all female, sterile, eyed eggs, and the resulting fish must be maintained in a freshwater, land based, physically contained facility [1].

The founding line of AquAdvantage^® ^Salmon was created in 1989, and an Investigational New Animal Drug (INAD) dossier was opened with the FDA in 1995. In 2009, AquaBounty submitted to the FDA the last of more than 25 scientific studies in support of our application. In 2010 the FDA presented their findings to the Veterinary Medical Advisory Committee (VMAC), and recommended an approval. The VMAC committee, comprised of independent experts selected from academia, industry, and NGO's, concurred with the FDA's findings, which were that AquAdvantage^® ^Salmon is equivalent to conventional farmed Atlantic salmon, and is safe for human consumption as well as safe for the environment. The regulatory analysis included an environmental assessment of a proposed AquAdvantage^® ^Salmon egg production site in Canada, and an AquAdvantage^® ^Salmon growout site in Panama, both of which were inspected and approved by the FDA. Important biosecurity elements for containing AquAdvantage^® ^Salmon and minimizing potential risk to the environment at the two production sites include physical, biological, and environmental containment. For example, at the proposed growout site in Panama which is more than 120 km from the nearest ocean, sterile, all female populations of AquAdvantage^® ^Salmon are confined to a highly biosecure culture facility possessing 21 individual containment elements, with a minimum of eleven containment barriers in sequence. In addition to the biological and physical containment elements, a natural thermal barrier of lethally high water temperatures (for Atlantic salmon) downstream of the facility would effectively prevent any live, escaped AquAdvantage^® ^Salmon from reaching the Pacific Ocean.

In 2012 the FDA published the environmental assessment for public commentary, and a Finding of No Significant Impact (FONSI), indicating that rearing AquAdvantage^® ^Salmon in those sites did not present a risk to the environment when reared under the conditions of use stipulated by the FDA. The public comment period concluded in April of 2013, and a final resolution of the application is expected this year from the FDA. If approved by the FDA, the product offered by AquaBounty would be eyed eggs of AquAdvantage^® ^Salmon, which would carry an FDA approved label indicating that they are female, triploid, genetically engineered Atlantic salmon eggs carrying a single copy of the AquAdvantage^®^gene construct. The label would also stipulate that the fish from these eggs could only be grown in pre-approved, land based, physically contained, freshwater aquaculture facilities.

As a pioneering application for a genetically modified animal destined for human consumption, AquAdvantage^® ^Salmon has attracted considerable attention and opposition by anti-GMO, anti-biotech activist organizations, as well as the organic food industry which is threatened by the more productive, lower cost, food production alternatives offered by GM plant and animal biotechnology. Resigned to accepting the inevitable regulatory approval of GM salmon and other innovative animal biotech products, the anti-GMO community is now desperately resorting to labeling initiatives, and economic extortion of future market stakeholders, in an effort to commercially stigmatize genetically modified food products.
